# Newborn Care in the Home and Health Facility: Formative Findings for Intervention Research in Cambodia

**DOI:** 10.3390/healthcare4040094

**Published:** 2016-12-21

**Authors:** Alessandra N. Bazzano, Leah Taub, Richard A. Oberhelman, Chivorn Var

**Affiliations:** 1 Department of Global Community Health and Behavioral Sciences, Tulane School Public Health and Tropical Medicine, New Orleans, LA 70112, USA; ltaub@tulane.edu (L.T.); oberhel@tulane.edu (R.A.O.); 2 Reproductive Health Association of Cambodia, P.O. Box 905, Phnom Penh, Cambodia; chivorn@rhac.org.kh; 3 National Institute of Public Health, P.O. Box 1300, Phnom Penh, Cambodia

**Keywords:** neonate, qualitative research, Southeast Asia

## Abstract

Global coverage and scale up of interventions to reduce newborn mortality remains low, though progress has been achieved in improving newborn survival in many low-income settings. An important factor in the success of newborn health interventions, and moving to scale, is appropriate design of community-based programs and strategies for local implementation. We report the results of formative research undertaken to inform the design of a newborn health intervention in Cambodia. Information was gathered on newborn care practices over a period of three months using multiple qualitative methods of data collection in the primary health facility and home setting. Analysis of the data indicated important gaps, both at home and facility level, between recommended newborn care practices and those typical in the study area. The results of this formative research have informed strategies for behavior change and improving referral of sick infants in the subsequent implementation study. Collection and dissemination of data on newborn care practices from settings such as these can contribute to efforts to advance survival, growth and development of newborns for intervention research, and for future newborn health programming.

## 1. Background

Effective interventions to combat newborn illness and mortality at the community level in low-income settings are available and information on these has been widely disseminated [1]. Yet coverage and scale-up of these interventions is suboptimal, despite evidence for feasibility [2]. Thus, newborn mortality remains an important public health problem in many low-income countries, and may occur in settings where skilled care at delivery is still improving, and where under 5-year-old mortality and maternal mortality have already decreased [3,4]. Up to 44% of the under 5-year-old mortality globally occurs in the newborn period (during the first 28 days after birth) [5]. In order to address concerns over newborn mortality in Cambodia, which has been targeted, alongside maternal survival, as a high priority health issue by the national government [6,7], and to leverage recent improvements in newborn survival [8], community based interventions are being developed and tested in partnership with the Ministry of Health to provide evidence for scalable, locally appropriate strategies for sustainable progress.

Formative research presented here was conducted preliminary to the final design of an intervention study, in order to provide culturally and socially relevant information on newborn care in home and health facility settings. This data was intended for incorporation into a tailored approach to behavior change. The goal of the intervention study, the Newborn Infection Control and Care Initiative for health facilities to accelerate reduction of newborn mortality (NICCI) is to contribute to the reduction of newborn mortality and morbidity by addressing infection control in first-line Health Centers during the perinatal period, improving recognition of newborn illness, and facilitating prompt referral to appropriate care at the community and facility level [9].

Formative research has been demonstrated to be important for the design of effective, community-based interventions for newborn health, and for their adoption into local and national policy [10,11,12,13]. The qualitative study detailed here aimed to collect data relevant to intervention design and to local, national and international stakeholders. The key hypotheses of the qualitative research carried out prior to the implementation of the intervention were that (1) barriers to optimal newborn care in the perinatal period exist, including problems around infection control; (2) maternity care staff in Health Centers would benefit from refresher training on essential newborn care; (3) mothers of newborns and their families may find it difficult to recognize danger signs of newborn illness; and (4) barriers exist to care seeking for newborns in the community.

Using international guidelines on newborn care and postnatal care of mothers and newborns, as well as a review of the literature, we developed a framework for analysis of the results of formative data based on comparison with recommended essential newborn care practices and actions [14,15,16]. The full framework is available in Annex 1 and summarizes key recommendations covering the period around delivery in Health Centers as well as guidelines for care of the newborn at home, recognition of danger signs and care seeking (with advice for referral). These topical areas correspond with the data outlined below under the Results sections: Newborn care around the time of birth in health centers, Health facility conditions around newborn care, Newborn care practices at home following delivery, Danger signs, Care seeking and referral. Key reference documents from which the analytic framework was derived include the Essential Newborn Care Course [14]; Pregnancy, Childbirth, Postpartum and Newborn Care: a guide for essential practice [17]; and WHO Recommendations on Postnatal care of the mother and newborn [18].

## 2. Materials and Methods

Takeo, Cambodia where the study was conducted is typical of a rural, lower income setting in Southeast Asia—most inhabitants rely on small scale agricultural production and are Buddhist. However, as noted in the previous section, Cambodia has prioritized reduction of maternal and newborn mortality and, as such, facility based delivery has become the norm. The study methodology has been described in more detail elsewhere [19]. In brief, qualitative formative research was carried out from February through April of 2014 to collect data based on a rapid ethnographic approach. Research was conducted in five operational districts in Takeo Province: Ang Rokar, Bati, DaunKeo, Kirivong, Prey Kabass. The region is located in the southwestern part of the country, with the province’s southern border abutting Vietnam. Data were collected from observation sessions (in homes and Health Centers where infants are delivered), interviews, and focus groups discussions (FGD), outlined in Table 1. Study participants included mothers and caretakers of children under 2 years old, Health Center midwives, and community health volunteers (known in Cambodia as Village Health Support Group volunteers or VHSG), and photos and video were collected during observations. Purposive sampling was used to identify participants representative of the study population. To identify VHSG volunteers for participation, the researchers contacted staff of a local non-governmental organization (NGO) working with VHSGs in the area and selected participants on the principle of maximum variation in sociodemographic characteristics and years of experience. For the selection of mothers/caretakers of young children, researchers asked VHSG volunteers (who work closely with community members in Health Center catchment areas) to assist in identifying potential participants from their communities based on characteristics of interest for the study, such as distance from home to health facility, and sociodemographic characteristics such as age, ethnicity, occupation and parity.

The data collection team consisted of five researchers, including the first author, experienced in qualitative research, and four junior social science researchers. Topic guides were used for interviews and FGDs and a checklist was used for observation sessions. All interviews and FGDs were audio recorded, and field notes were translated to more detailed transcription notes. Audio recordings were also used to check the quality of field notes.

Ethical approval for this study was obtained from the Cambodian National Ethics Committee for Health Research and reviewed by the Institutional Review Board of Tulane University Medical Center. Informed consent was obtained from all study participants by signature or thumbprint.

Two researchers participated in analysis of the data and discussed findings throughout the process to reach consensus. The analytic approach, was based on a constant comparative method of identifying concepts and phenomena and assessing their relation to emergent themes [20], guided by the analytic framework derived from the literature on recommended newborn care practices. In the analysis we applied existing frameworks for newborn care but also developed inductive codes through the constant comparison method. All data were analyzed both in Microsoft Word and in NVivo Version 10 (QSR International Pty Ltd., Melbourne, Australia).

## 3. Results

The formative research identified gaps between recommended neonatal care practices, based on international guidance for essential newborn care, and those recorded in the study areas. These gaps were identified around the time of birth in the Health Centers where women delivered, and during the first month of life in the home and community setting. In addition, health facility practices, particularly around infection control, that could impact newborn health were noted. Several domains of interest were identified.

### 3.1. Newborn Care around the Time of Birth in Health Centers

Methods of newborn care immediately after delivery in Health Centers varied little among study participants. Women and midwives alike reported that the newborn was placed on the abdomen of the mother while the cord was cut with clean birthing equipment. Participants also overwhelmingly reported that newborns were dried with a sarong provided by the mother from home, and then wrapped in the same (wet) sarong that had been used for drying. In only a couple of instances were newborns reported to be dried using clean, dry clothes from the Health Center (most facilities did not have linens available for the purpose of drying).

One midwife explained the process of birth this way:

If I am by myself, I would take the newborn, hand it to the mother to hold on her abdomen and then I would clean the face, the space around the eyes, inject oxytocin to the mother to make the placenta come out and then clamp the umbilical cord. Then I will clean the baby and weigh them before wrapping. The same cloth (sarong) for wiping and cleaning can be used to wrap the baby after.

*(Primary midwife, 24 years old, Takeo Province)*

Midwives reported frequent shortages of gloves and using the same pair of gloves from the start of delivery through post-partum care. They did not report any training or practice on changing gloves or washing gloved hands prior to attending to the newborn. Participants reported that the newborn was placed on the mother’s abdomen after delivery while midwives attended to the placenta and cord, but skin to skin contact after the delivery was complete (lasting for at least 2 h postpartum per international guidelines) was not reported by women to be usual practice and it was not observed during any of the visits with participants.

Midwives reported advising mothers to exclusively breastfeed soon after delivery, but participants did not report initiating breastfeeding immediately following birth. During observations at health facilities, mothers were not seen breastfeeding their newborns, and midwives were not observed counseling or discussing breastfeeding with mothers. In one health center where a birth had taken place less than thirty minutes prior to observation, a midwife said that she did not feel comfortable asking the new mother to breastfeed. Some midwives, particularly those who were younger in age, or were not mothers themselves, stated that they were uncomfortable advising mothers in detail on breastfeeding.

At Health Center level, one midwife reported:

If the baby is weak then they cannot breastfeed. Sometimes, the baby can’t breastfeed because of problem with mom’s breast. They give formula milk in this case. The mother buys the formula. The mother and the grandmother prepare.

*(Primary midwife, 26 years old, Takeo Province)*

Based on observations and midwives’ reports of usual practice following delivery, midwives typically did not assess newborn health post-partum, though mothers’ vital signs and health were assessed in the post delivery room.

I advise them not to put anything on the cord except for betadine (povidone iodine). I have heard that some of them use the insect nests, spider nest, ash, or other things on the cord. But other than that I don’t really advise them on how to take care of the newborn.

*(Primary midwife, 24 years old, Takeo Province)*

Midwives reported familiarity with danger signs of newborn illness, but stated that newborn illnesses at birth were very rare, mostly related to respiratory infections in the study area, and would be treated at the referral hospital after discharge from the Health Center.

It is rare for them because after the baby is born, after 1 day they go home. So if they are sick they might go to the referral hospital.

*(Primary midwife, 26 years old, Takeo Province)*

When asked whether mothers bring in ill newborns shortly after delivery, one participant responded this way:

Very few (come for illness), such as the eye. A few, their umbilical cord after the cord drops is not cured yet and they bring it here. The babies are brought to the OPD (Outpatient Department).

*(Primary midwife, age unknown, Takeo Province)*

Regarding typical post-natal care for newborns, one midwife described this:

If there is no problem the newborn goes home. A few days later they come for BCG (Bacillus Calmette-Guerin immunization). One and a half months later come for immunizations. Health center only gives BCG on Mondays so if baby is not born on Monday then it has to wait a few days to receive BCG.

*(Primary midwife, 44 years old, Takeo Province)*

When asked about low birth weight infants and special care for them in the Health Center midwives responded this way:

Yes we have some, but not dying. Out of a hundred, maybe 5–6 (are low birth weight). They survive. From time to time there is a death. One woman had 10 children and the baby was weak and could not breastfeed. There’s another case also, of underweight. The first 3 days the baby was ok. But on the 4th day after baby’s bath, the baby stopped breathing. A fire was lit below the bed and newborn was wrapped to keep it warm so it survived.

Mothers don’t know that low birth weight babies need special care. Sometimes we tell them but they still want to give baths to the baby.

Low birth weight newborns easily get cold body temperature and then stop breathing. Aside from temperature they have cough, fever, runny nose diarrhea.

*(Focus Group Discussion with Midwives 28–46 years old, Takeo Province)*

### 3.2. Health Facility Conditions around Newborn Care

Numerous issues were identified around delivery and postnatal care in Health Centers. Many Health Centers lacked sufficient supportive infrastructure (e.g., steady supply of electricity, availability of backup electricity, sufficient lighting) to ensure healthy newborn care. One midwife described the situation this way:

The post delivery room building is too old. Even if we try to clean it’s not clean. Also, (it is) too small. Can only fit 2 mothers. If there are 3, then one has to stay outside.

*(Primary midwife, 46 years old, Takeo Province)*

**Figure 1:**
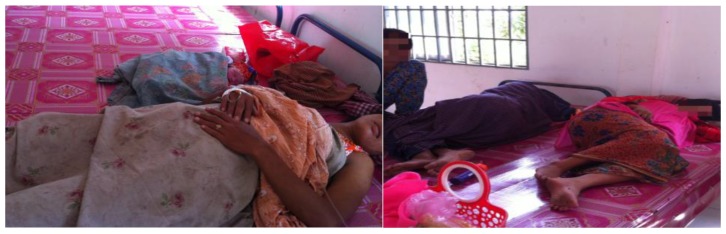
Health facility postnatal area.

Post-delivery beds where newborns and mothers rested were not reported as cleaned by health staff, with sleeping mats and bed coverings being reused. The photos in Figure 1 show a post-delivery area for newborns and mothers to recover.

Waste receptacles for safe disposal of consumables (e.g., sanitary pads, diapering supplies, food remnants and containers) were absent in postnatal areas. Waste bins observed on visits were sometimes overflowing and dusty in labor and delivery rooms. Latrines used by mothers and families, and for safe disposal of infant feces, and were found to be unsanitary and inadequate in number throughout the observations in the study area. Many latrines were located far from labor and delivery areas and lacked stations for hand washing.

Although midwives universally said that they practiced hand washing, researchers did not observe any hand washing during Health Center visits. Many circumstances were identified that impeded frequent and effective hand washing, including a dearth of functional hand washing stations near areas where health care was carried out, and lack of soap and towels between hand washing points and latrines. There were also several water-related barriers to hand hygiene during health facility observations. Indoor water and sinks did not always function properly, and outdoor running water was sometimes unavailable. Emergency stores of water kept in large ceramic containers appeared cloudy and foul smelling, or were missing covers, exposing water to environmental pollution and creating a breeding ground for vectors. In almost all Health Centers, post-partum women and their families did not appear to have access to hand washing stations, as there were often no hand washing stations available. While most Health Centers had soap available, only one Health Center had towels, which were not necessarily cleaned regularly, for drying hands.

Midwives said that during instances in which they forgot to wash their hands due to time constraints, they relied on gloves to protect the patient and provider. The use of two pairs of gloves (non-sterile and sterile) was seen as protection equivalent to or better than hand washing. They said that in instances where there were no gloves for delivery, families or health staff were required to purchase gloves from the market.

This month there were fewer deliveries, 13 only, but in some months with many more, there is a shortage of gloves… If there is low supply the health center buys from the market.

*(Primary midwife, 46 years old, Takeo Province)*

Most Health Centers did not have dedicated staff for cleaning labor and post-delivery rooms. Instead, midwives who carried out deliveries were deemed responsible for cleaning.

Many midwives reported that they either requested the families of women who delivered at the Health Center to clean, or were unable to do so adequately, if at all, due to the heavy burden of patient care.

**Figure 2:**
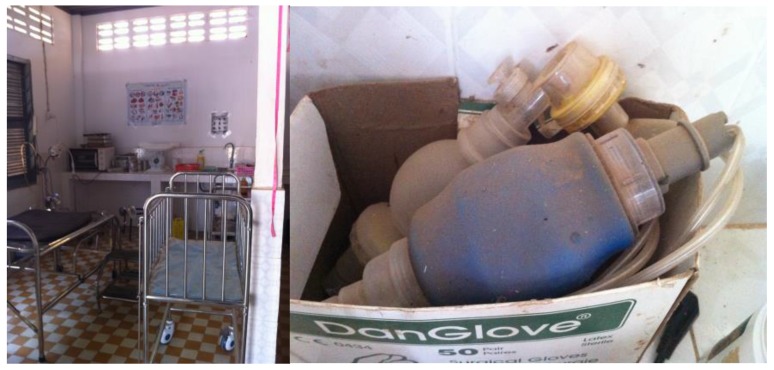
Health facility delivery room and bag valve masks for resuscitation.

Cleaning supplies, along with instruments and equipment for perinatal care at Health Centers appeared in suboptimal condition on observation visits. Cleaning implements and products were haphazardly stored or unavailable. In addition, for example, resuscitation equipment was not well maintained (see Figure 2) within delivery rooms.

Dish soap was the most commonly observed and reported cleaning agent for surfaces the newborn could contact, delivery room floors and operating tables. Some Health Centers had bleach or chlorhexidine, but staff did not report using these products for routine cleaning or were unable to recall the exact procedures for disinfection. One focus group discussion elicited confusion on the matter:

We do not have any spray to kill the germs (in the delivery and post-delivery rooms) but we use soap, “eau de javel” [liquid bleach].

*(Primary midwife, 28 years old, Takeo Province)*

But now “eau de javel” [liquid bleach] is not allowed, we were told to use dishwashing soap.

*(Primary midwife, 46 years old, Takeo Province)*

We do not use bleach as of now.

*(Primary midwive, 44 years old, Takeo Province)*

Linens used for newborn care around the time of delivery were also identified as a potential problem. A few Health Centers maintained linens for two purposes: white cloths to dry and place under newborns during weighing, and green cloths to wrap sterilized instruments. In all other instances, the family of the woman delivering was responsible for providing linens (e.g., sarongs, diapers, dressing gowns or blankets, sanitary napkins, towels to dry newborns and hands). However, it was unclear how midwives or families washed or dried these linens in the Health Center with no washing facilities available to families.

### 3.3. Newborn Care Practices at Home Following Delivery

While mothers, caregivers, VHSGs and midwives universally reported that newborns were exclusively breastfed for the first six months, observations and probing revealed that major gaps existed in recommended breastfeeding practices. Women observed and interviewed did not receive detailed instruction on breastfeeding from midwives around the time of birth, no in-depth counseling for problems, and typically stayed in the Health Center for less than 24 h before returning home.

During home observations and interviews, women were observed breastfeeding newborns. Problems with latch and positioning of infants were noted in two cases, and feeding sessions lasted less than ten-minutes (presence of observers may have impacted this). Mothers typically used a scissor hand position to present the breast to newborns, compressing the front of the breast and nipple area. Mothers also placed newborns on their backs and turned away from their own body to position for breastfeeding. In two cases, the mothers also displayed a high degree of pain while breastfeeding. One mother described her experiences:

I breastfed one day after the birth. The baby cried a lot when I first started breastfeeding with colostrum. I bought canned milk to give the baby. When the white milk came in I then gave the baby only the breast milk. I had problems breastfeeding. Nipples became cracked, I felt pain. I did nothing, just endured the pain and kept breastfeeding.

*(Mother, 26 years old, housewife, Takeo Province)*

Women and older female VHSGs reported that newborns were fed with only one breast per feeding session in order to maintain breast shape. The impact of breastfeeding on the appearance of breasts was described as a concern. One mother reported:

Women don’t want their breasts to be out of shape. If they can afford formula, some won’t breastfeed baby.

*(Mother, 36 years old, housewife, Takeo Province)*

In homes, newborn feeding bottles were often observed. Those observed were empty or filled with water, even where the mother reporting that the newborn was exclusively breastfed. In many homes, infant formula was also present alongside the bottles, and many participants reported feeding infants formula or other breast milk substitutes (e.g., canned milk). For many women, their family members (mothers and sisters) were their main source of advice on breastfeeding practices.

**Figure 3:**
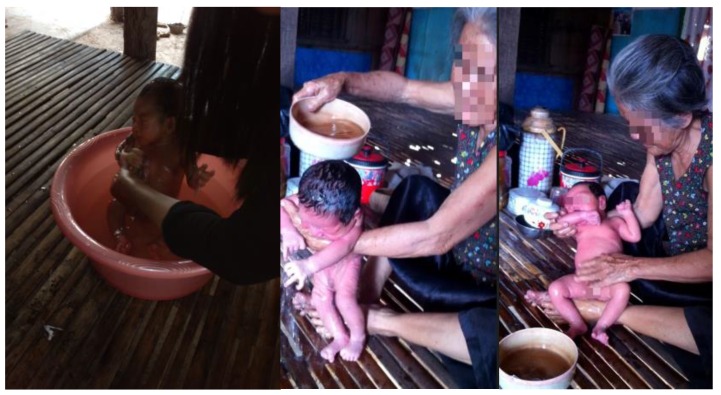
Bathing of newborns.

The most common practice for bathing of newborns (both observed and reported during interviews) was outdoor bathing using a plastic tub with tepid water. Families reported bathing newborns 2–3 times during the day. The reason given for this practice was warm environmental temperatures causing discomfort for the newborn. Grandmothers or older female family members were typically responsible for bathing the newborn and used lukewarm water. Figure 3, Illustrates typical scenes of newborn bathing.

According to one mother:

To bathe the baby the grandmother puts the newborn in a container with warm water. The water is then poured over the baby. We did not use soap to bathe the baby because I was afraid of the baby having an allergic reaction since the baby’s skin is “young.”

*(Mother, 27 years old, housewife, Takeo Province)*

Warm water for bathing was observed to be stored in containers such as plastic jugs or thermoses. In one instance, the water used for bathing appeared cloudy and the newborn could be seen drinking it (see Figure 3). Families described and were observed in a few instances mixing boiled water with cold water. In two observations of bathing, newborns were noted to be shivering due to temperature fluctuation. A few mothers reported bathing with special liquid baby soaps, which were thought to be milder for the infant’s skin.

**Figure 4:**
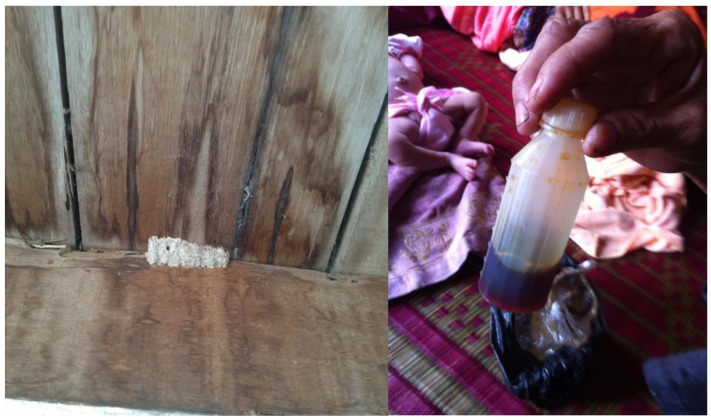
Substances applied to the umbilical cord.

Various substances were reported and observed to be applied to newborn umbilical cords (see Figure 4 below). One such substance consisted of insect nests, an ash-like material commonly found in the eaves of wooden Cambodian homes (photo available on request). This was reported to be an older, traditional practice and described as being applied to the newborn umbilicus daily until the cord fell off. Another substance more commonly applied and given to families (or recommended) at Health Centers was betadine, or povidone iodine liquid. In one home, betadine was observed to be applied to the newborn cord by a grandmother. The bottle of betadine was dirty, dusty and kept in a bag along with a piece of gauze used repeatedly to apply the betadine to the cord.

During home visits, diapering practices were observed and documented. Typically, the newborn was dressed in a small cloth lined with a paper towel, used after bathing and continuously throughout the day and night. Families also often had commercial baby wipes to clean the baby’s bottom, which were often made in China and the ingredients of which were not clearly labeled.

Participants in all study sites reported that they applied various medicinal balms to the abdomen and/or fontanel of the newborn. This was perceived as important for toughening or hardening the skin of the newborn to prevent future illness. Various brands of balm were observed, and most contained menthol or other “hot” ingredients perceived to be beneficial for newborns. Baby powder was also frequently used for newborn skin care after bathing. Caregivers did not report applying emollients or lotions, though baby lotions were observed in two homes.

Environmental conditions outside participant homes were often not hygienic. In most cases, refuse was not containerized and was distributed around the perimeter of homes. Domesticated animals were kept in close proximity to eating, sleeping and resting areas. Most homes did not have latrines. Families without latrines described using neighbors’ latrines or nearby fields for elimination. Children (under eight years) were observed and reported to defecate along the road or near fields close to homes. Water was present in all homes, but varied by source and quality. Many families noted significant problems accessing clean water despite having a well nearby or on their property. In some areas, water was purchased for cooking and drinking, while water for washing was sourced from dirty and brackish runoff or ponds with rainwater (photos available as Appendix Table A1).

### 3.4. Danger Signs

Mothers and VHSGs were familiar with the newborn danger signs of fever and refusal to breastfeed, but were generally not able to name any others. A few mentioned hearing about convulsions; however, one mother perceived convulsions as normal.

I have heard about and seen convulsions in other babies. It happens after giving birth. With the convulsion I witness, nothing was done as the family believed it’s a natural thing. Let the baby be. It was born like this.

*(Mother, 26 years old, housewife Takeo province)*

This lack of awareness of dangers signs extended to informants with relatively high educational attainment and socioeconomic status. One father, whose wife was a secondary school teacher, relayed his experience around the birth of his low birth weight baby.

At the hospital we did not receive any special instructions on how to take care of the small baby (or any instructions at all on any newborn care). I did not think there was any special care for smaller babies versus normal babies, only just did whatever came to my mind to care for the baby. I stayed for 1 full week at the Hospital. The doctor gave my wife some pills to take after the birth. While staying at the Hospital, I did feel that if I called them they would come and help with the baby. I did call for help when it seemed to me that the baby had “short of breath”. I thought that something was caught in her throat or she was choking, so I called them and the staff suctioned the baby’s throat. She continued to be “short of breath” all the while they stayed in the hospital.

*(Father, 30 years old, farmer, Takeo province)*

### 3.5. Care seeking and Referral

Participants reported different care seeking patterns for newborn illness depending on severity. Mothers stated that hypothetically they would take a sick newborn to the nearest health provider for assessment. In many cases, this would be a private provider, “cabinet”, or pharmacy located in the immediate village vicinity (e.g., for paracetamol to treat fever). In other cases the nearest choice was the local Health Center. A few caretakers mentioned that they would always take the newborn to the Health Center first because it was inexpensive or free, or because the infant was born there.

I would go to the Health Center if baby was sick. The reason is that it is close to his home, also it is cheap. If the baby wasn’t cured for a few days after the Health Center visit and getting more serious, I would go to the private doctor.

*(Father, 30 years old, farmer, Takeo province)*

However, families said that in very serious cases of newborn illness they would go directly to a referral level hospital, either in Takeo Province or Phnom Penh, bypassing any referral from primary providers.

If my baby got sick, I will go to somewhere near the market, there is a private cabinet. Not to the Health Center, because I don’t need to wait at the private cabinet, and I believe that it is more effective to see in private. It costs me 5000 to 10,000 riels in the private cabinet, and only 1500 riels at the Health Center. If my baby gets seriously sick I would go straight to Kantha Bopha Hospital in Phnom Penh because I was told that at Kantha Bopha Hospital the services is very good, the quality of the doctors also good and the medicine is also effective to cure the baby illness. I don’t think there is someone specialized in newborn illness around here.

*(Mother, 23 years old, housewife, Takeo Province)*

For care seeking, mothers usually go to the nearest place. Usually they would go to the Health Center because as mothers we may not know the diseases of babies but they at the Health Center are experts so we will go there. The reason to go to another place than the Health Center is if the other place (such as a cabinet) is very near to the home, nearer than the Health Center. I know Takeo Referral Hospital and I would go there if my baby was severely ill but so far I have never been there. I would go there instead of Phnom Penh because it is closer.

*(Mother, 29 years old, housewife, Takeo Province)*

Midwives reported treating very few cases of newborn illness. Although some drugs were identified by staff as used for newborn illness (powdered ampicillin for oral treatment, paracetamol syrup, and promethazine syrup for cough), midwives did not report much experience or familiarity with treating newborns.

## 4. Discussion

Evidence-based recommendations on care of the baby at the time of birth, such as those provided by WHO [17] outline important practices in four core areas: hygiene (protection), breathing, temperature control (warmth), and feeding. The current study highlighted many gaps in practices in these areas that may impact on newborn survival, growth and development, and provided potential behavior change targets (outlined in Table 2 below). Care seeking is another area of concern in the study area, and has been identified as a problem related to newborn survival in many low-income settings [21].

Recent interventions have shown promise in improving newborn health through community and facility based approaches [13,22,23,24]. In the study setting, a linked community and facility based intervention is currently underway, which may address some of the identified barriers to newborn survival [9].

An important point of care for newborns is comprehensive assessment before release from the Health Center, which appears to be sub-optimal in this setting and is an important omission related to identification of ill newborns or those at risk. Midwives did not report assessment of the newborn prior to discharge as a standard practice, and it is unlikely that they actively look for danger signs in the crucial twenty-four hours after birth. Postnatal care in Cambodia has been highlighted as requiring improvement [25] and an important area to address for newborn health. In other settings, improvements in essential newborn care have made an important difference in improving health [26].

The importance of appropriate wrapping and drying for thermal control of newborn infants has been demonstrated in other studies [27,28]. In the study area, the practice of wrapping the baby immediately after birth in the same cloth used for drying is a practice likely to increase the risk of hypothermia and infection in newborns. Barriers and facilitators to appropriate care in this area are similar to those found in other settings [16,29,30].

One option for improving thermal control is the evidence-based practice of appropriate skin-to-skin care [31,32,33]. The study identified barriers to this practice in this setting. Firstly, knowledge on the part of health staff may be low. Providers understand that they should place the newborn on the mother’s abdomen during cord cutting or immediately after delivery, but they did not report the practice of keeping a newborn and mother in skin-to-skin contact beyond those few minutes in the delivery room. A second possible barrier is related to women’s preferences and lack of awareness of the benefits of skin-to-skin contact. Women were observed wearing warm clothing around the time of delivery and particularly post-partum. This is likely related to the local understanding of post-partum as a time when women need to be kept very warm for recovery. It would follow that women are unwilling to have their upper bodies exposed in order to carry out skin-to-skin contact (even with a blanket or towel to cover and warm both the mother and baby). A final consideration is that modesty may prevent women from carrying out skin to skin contact or from facility staff recommending and encouraging it beyond the immediate period in the delivery room.

Insufficient breastfeeding advice and support for women is also a barrier to improving newborn health in this setting. The UNICEF Baby Friendly Hospital Initiative Training Course indicates that facility staff need to go beyond verbally advising women to breastfeed in order to provide lactation support, and this issue has been detailed in another study [34,35].

A recent multi-country analysis of bottlenecks related to newborn care identified several bottlenecks at the health system [36]. Many of these, such as health workforce bottlenecks and service delivery bottlenecks, are found in the study setting. Interventions that improve the ability of health providers to deliver care, and to provide guidance to families on basic newborn care, are likely to have a beneficial impact.

Strengths and Limitations: Strengths of the study included the ability to understand several care practices in depth and to describe the complex issues around newborn care. We have also been able to provide information from the perspective of the participants, in many cases in their own words. The study limitations include those typical of qualitative studies in health research such as a small number of observations and data points, as well as the inability to generalize findings broadly beyond the study setting. Another important limitation was the restriction to studying only infants born in primary health facilities, which may have missed practices important to newborn health that occur in home deliveries, or to those in higher level health facilities such as referral hospitals.

## 5. Conclusions

Through field observations, interviews and focus group discussions, the research identified several practices that do not align with evidence-based recommendations on newborn care and are key targets for intervention. Health facility problems that may present a barrier to appropriate newborn care at birth were also identified. Based on this formative research, topical areas for behavior change were prioritized. Results of the intervention underway in the study area will provide additional information on the links between essential care practices and newborn health.

## Figures and Tables

**Table 1:** Study methodology.

Inquiry	Semi Structured Interviews	Observation in the Home	Photo and Video Documentation	Focus Group Discussions
Data Collected	Perceived newborn health problems (including danger signs); Local understanding of illnesses; Newborn care practices; Care seeking behaviors; Referral practices	Newborn care practices	Hygiene and infection control practices in Health Centers and related equipment and supplies	Hygiene and infection control practices in Health Centers; Newborn care practices; Perceived newborn health problems (including danger signs) and referral
Participants	27 Mothers/Caregivers; 16 VHSG volunteers	4 newborns (less than 29 days old)	10 health facilities and 4 homes of newborn babies	2 FGDs (8 participants)

**Table 2:** Recommended areas for intervention.

Newborn Care at Health Center	Newborn Care at Home
Routine cleaning of delivery and labor rooms	Emphasizing dry cord care or chlorhexidine
Hand hygiene in HC	Limiting bathing and using clean, warm water
Clean dry cloth to wrap baby after delivery	Exclusive breastfeeding (no water or supplemental formula)
Encourage skin to skin contact following delivery	Hand hygiene at home
Postnatal assessment of newborn prior to discharge	Environmental hygiene at home
Improved counseling and support for breastfeeding initiation, timing/frequency of feeds, assessment of positioning and latch	Improved recognition of danger signs at community/household level
Guidance and counseling on newborn danger signs and care seeking	Clear referral processes for families to seek prompt and appropriate care for newborn illness

## Appendix

**Table A1:** Analytic framework of newborn care recommendations.

Newborn Care around the Time of Birth/Health Facility Conditions	Newborn Care Practices at Home Following Delivery
**Protection (Hygiene)**	
Standard precautions and cleanliness: Wash hands Wear gloves Protect yourself from blood and other body fluids during deliveries Practice safe disposal of sharps Practice safe waste disposal Deal with contaminated laundry Sterilize and clean contaminated equipment Sterilize gloves Change gloves (if possible) prior to cutting the cord. Have sterile kit to tie and cut cord (protection)	Wash your hands with soap and water before and after handling your baby, especially after touching her/his bottom. Wash hands before and after cord care. Put nothing on the stump. DO NOT apply any substances or medicine to stump. Wash baby’s bottom when soiled and dry it thoroughly. Practice safe disposal of infant feces and household waste.
**Warmth (Temperature control)**	
Have clean warm towels/covers/cloths ready for newborn baby at delivery. Thoroughly dry the baby immediately. Discard wet cloth. Skin-to-skin contact: Leave the baby on the mother’s abdomen (before cord cut) or chest (after cord cut) after birth for at least 2 h. Cover the baby with a soft dry cloth. Help mother to wear clothes which make immediate skin contact easy.	Keep baby warm and covered. Avoid frequent bathing, changes in baby’s temperature. Newborns need more clothing than other children or adults. If cold, put a hat on the baby’s head. During cold nights, cover the baby with an extra blanket.
**Breathing**	
Assess the newborn. Have resuscitation equipment near delivery bed. Keep equipment in good condition. Monitor the baby every 15 min after delivery.	Advise the mother to seek care for the baby as needed, to observe baby and note danger signs for care seeking, especially difficulty breathing, fast or slow breathing, grunting, or chest in-drawing.
**Feeding**	
Encourage immediate breastfeeding. Keeping mother and baby in skin-to-skin contact from birth encourages early breastfeeding. Counsel the mother on breastfeeding. Help the mother to initiate within one hour. Assess breastfeeding. Encourage breastfeeding on demand, day and night, as long as the baby wants. → A baby needs to feed day and night, 8 or more times in 24 h from birth. Only on the first day may a full-term baby sleep many hours after a good feed. → A small baby should be encouraged to feed, day and night, at least 8 times in 24 h from birth. Teach correct positioning and attachment. Show the mother how to hold her baby. She should: → make sure the baby’s head and body are in a straight line → make sure the baby is facing the breast, the baby’s nose is opposite her nipple → hold the baby’s body close to her body → support the baby’s whole body, not just the neck and shoulders Show the mother how to help her baby to attach. She should: → touch her baby’s lips with her nipple → wait until her baby’s mouth is opened wide → move her baby quickly onto her breast, aiming the infant’s lower lip well below the nipple. Look for signs of good attachment and effective suckling (that is, slow, deep sucks, sometimes pausing). If the attachment or suckling is not good, try again. Then reassess	Advice for mother: Start breastfeeding within 1 hour of birth. The baby’s suck stimulates your milk production. The more the baby feeds, the more milk you will produce. Give your baby the first milk (colostrum). It is nutritious and has antibodies to help keep your baby healthy. At each feeding, let the baby feed and release your breast, and then offer your second breast. At the next feeding, alternate and begin with the second breast. Exclusive breastfeeding for the first 6 months. Seek care for problems around breast feeding. Immediately after birth, keep your baby in the bed with you, or within easy reach. At night, let your baby sleep with you, within easy reach. While breastfeeding, you should drink plenty of clean, safe water. You should eat more and healthier foods and rest when you can.
**Danger signs**	**Care seeking and referral**
Stopped feeding well, history of convulsions, fast breathing (breathing rate ≥ 60 per minute), severe chest in-drawing, no spontaneous movement, fever (temperature ≥ 37.5°C), low body temperature (temperature < 35.5°C), any jaundice in first 24 h of life, or yellow palms and soles at any age. The family should be encouraged to seek health care early if they identify any of the above danger signs in between postnatal care visits.	Return or go to the hospital immediately if the baby has: difficulty breathing, convulsions, fever or feels cold, bleeding, diarrhea, very small, just born, not feeding at all. Go to the Health Center as quickly as possible if the baby has: difficulty feeding, pus from eyes, skin pustules, yellow skin, a cord stump which is red or draining pus, feeds <5 times in 24 h. During transportation: Keep the baby warm by skin-to-skin contact with mother or someone else. Cover the baby with a blanket and cover her/his head with a cap. Protect the baby from direct sunshine. Encourage breastfeeding during the journey. If the baby does not breastfeed and journey is more than 3 h, consider giving expressed breast milk by cup.
